# A Gain-of-Function Mutant of IAA15 Inhibits Lateral Root Development by Transcriptional Repression of *LBD* Genes in Arabidopsis

**DOI:** 10.3389/fpls.2020.01239

**Published:** 2020-08-12

**Authors:** Sun Ho Kim, Sunghwa Bahk, Jonguk An, Shah Hussain, Nhan Thi Nguyen, Huy Loc Do, Jae-Yean Kim, Jong Chan Hong, Woo Sik Chung

**Affiliations:** Division of Applied Life Science (BK21 Plus Program), Plant Molecular Biology and Biotechnology Research Center, Gyeongsang National University, Jinju, South Korea

**Keywords:** auxin, *Aux*/*IAA*, gain-of-function, lateral root, *LBD* genes, repressor

## Abstract

Lateral root development is known to be regulated by Aux/IAA-ARF modules in *Arabidopsis* *thaliana*. As components, several Aux/IAAs have participated in these Aux/IAA-ARF modules. In this study, to identify the biological function of IAA15 in plant developments, transgenic plant overexpressing the gain-of-function mutant of IAA15 (IAA15^P75S^ OX) under the control of dexamethasone (DEX) inducible promoter, in which IAA15 protein was mutated by changing Pro-75 residue to Ser at the degron motif in conserved domain II, was constructed. As a result, we found that IAA15^P75S^ OX plants show a decreased number of lateral roots. Coincidently, *IAA15* promoter-GUS reporter analysis revealed that *IAA15* transcripts were highly detected in all stages of developing lateral root tissues. It was also verified that the IAA15^P75S^ protein is strongly stabilized against proteasome-mediated protein degradation by inhibiting its poly-ubiquitination, resulting in the transcriptional repression of auxin-responsive genes. In particular, transcript levels of *LBD16* and *LBD29*, which are positive regulators of lateral root formation, dramatically repressed in IAA15^P75S^ OX plants. Furthermore, it was elucidated that IAA15 interacts with ARF7 and ARF19 and binds to the promoters of *LBD16* and *LBD29*, strongly suggesting that IAA15 represses lateral root formation through the transcriptional suppression of *LBD16* and *LBD29* by inhibiting ARF7 and ARF19 activity. Taken together, this study suggests that IAA15 also plays a key negative role in lateral root formation as a component of Aux/IAA-ARF modules.

## Introduction

Lateral root development is an auxin-regulated developmental process that maximizes the ability of the root system to absorb water and nutrients from the soil (Péret et al., 2009). The phytohormone auxin has been known to promote lateral root development (Torrey, 1950; Blakely et al., 1988). Exogenous auxin promotes cell division in the root pericycle, thereby inducing lateral root formation, whereas inhibitors of auxin transport inhibit lateral root initiation (Casimiro et al., 2001; Alarcón et al., 2019). In Arabidopsis, lateral root development begins with the auxin-dependent local activation of pericycle cells at the two protoxylem poles (Beeckman et al., 2001; Casimiro et al., 2001; Casimiro et al., 2003). These pericycle-derived cells undergo a series of cell division and differentiation processes, leading to the formation of lateral root (Laskowski et al., 1995).

Auxin regulates almost every aspect of plant growth and development, including embryo patterning, root and stem growth, organ senescence, vascular differentiation, seed germination, and flower development (Gälweiler et al., 1998; Friml et al., 2002; Ellis et al., 2005; Weijers et al., 2006; Wang et al., 2013; Hussain et al., 2020). The auxin-signaling pathway is highly conserved in all higher plants (Reed, 2001; Guilfoyle and Hagen, 2007; Mockaitis and Estelle, 2008). Three components primarily control auxin signaling: the auxin receptors TRANSPORT INHIBITOR RESPONSE1 (TIR1)/AUXIN SIGNALING F-BOX PROTEINs (AFBs), transcriptional repressors AUXIN/INDOLE-3-ACETIC ACIDs (Aux/IAAs), and transcription factors AUXIN RESPONSE FACTORs (ARFs). Under low auxin levels, Aux/IAA proteins recruit the co-repressor TOPLESS to inhibit the transcription of auxin-responsive genes by repressing the activities of their interaction partners ARFs. ARFs directly bind to the promoters of their target genes and activate or inhibit their transcription (Weijers et al., 2005; Szemenyei et al., 2008). Under high auxin levels, auxin is perceived by the auxin receptors TIR1/AFBs, and the SCF^TIR1/AFBs^ E3 ubiquitin-ligase complex targets Aux/IAA repressors for degradation *via* poly-ubiquitination (Dharmasiri et al., 2005; Mockaitis and Estelle, 2008). Once Aux/IAAs are degraded by the 26S proteasome, the free ARFs initiate the transcription of auxin-responsive genes (Ulmasov et al., 1997; Weijers et al., 2005).

The *Aux*/*IAAs* family in Arabidopsis were first identified based on their strong induction by auxin at the transcriptional level. Most Aux/IAA proteins contain four conserved domains (I, II, III, and IV) (Liscum and Reed, 2002). Domain I contributes to the transcriptional repression of Aux/IAA targets by recruiting the co-repressor, TOPLESS. Domain II contains a highly conserved degron motif (VGWPPV), the docking site of the auxin-activated SCF^TIR1/AFBs^ ubiquitin E3 ligase complex, leading to the poly-ubiquitination of the target proteins. Domain III and IV are involved in homo- or hetero-interactions with other Aux/IAAs or ARFs. When the conserved amino acid in domain II of an Aux/IAA protein harbors a point mutation, the protein becomes stable because it cannot interact with SCF^TIR1/AFBs^, even in the presence of auxin. Stabilized Aux/IAA mutants are thought to constitutively interact with and inactivate ARFs during various biological processes, thereby inhibiting auxin signaling. Many biological functions of Aux/IAAs have been identified by investigating various gain-of-function mutants of *Aux*/*IAA*, such as *iaa1*/*axr5*, *iaa3*/*shy2*, *iaa6*/*shy1*, *iaa7*/*axr2*, *iaa8*, *iaa12*/*bdl*, *iaa14*/*slr*, *iaa16*, *iaa17*/*axr3*, *iaa18*, *iaa19*/*msg2*, and *iaa28* (Kim et al., 1996; Rouse et al., 1998; Tian and Reed, 1999; Nagpal et al., 2000; Rogg et al., 2001; Hamann et al., 2002; Tatematsu et al., 2004; Yang et al., 2004; Fukaki et al., 2006; Ploense et al., 2009; De Rybel et al., 2010; Rinaldi et al., 2012; Wang et al., 2013). These mutations result in auxin-related phenotypes, including altered developments of hypocotyl, root, leaf, embryo, and tropisms.

Many genetic studies using Arabidopsis mutants have reported that lateral root development is primarily regulated by several *Aux*/*IAAs*, *ARFs*, and *LATERAL* *ORGAN BOUNDARIES*-*DOMAINs* (*LBDs*). For example, gain-of-function mutants of *Aux*/*IAA*, such as *iaa3*/*shy2*, *iaa14*/*slr*, *iaa19*/*msg2*, and *iaa28*, show severely reduced lateral root phenotypes due to inhibited ARF activity (Tian and Reed, 1999; Rogg et al., 2001; Tatematsu et al., 2004; Fukaki et al., 2006). Analysis of the *arf7*, *arf19*, and *arf7* *arf19* mutants showed that ARF7 and ARF19 play positive roles in lateral root formation (Okushima et al., 2005; Wilmoth et al., 2005). Furthermore, *LBD16* and *LBD29* play a positive role in lateral root formation downstream of ARF7 and ARF19 (Okushima et al., 2007; Goh et al., 2012). These studies provided the molecular signaling pathway of how Aux/IAA-ARF modules-mediated auxin signaling is involved in lateral root formation.

It was reported that IAA15 modulates auxin homeostasis that controls gravitropic responses and stem cell differentiation (Yan et al., 2013). However, the biological functions of IAA15 in other developmental processes were not extensively studied. In the current study, we generated transgenic Arabidopsis plants overexpressing a gain-of-function mutant form of IAA15 (IAA15^P75S^ OX) with a mutation in a conserved amino acid in domain II. The IAA15^P75S^ OX plants showed auxin-deficient phenotypes, including shortened primary roots and decreased lateral roots compared to the wild type. When we investigated the expression of *IAA15* gene by using *IAA15_pro_*::*GUS* reporter construct, *IAA15* transcripts were mainly detected in lateral root primordia and mature lateral root tips. Stabilized IAA15^P75S^ mutant protein inhibits the transcription of auxin-responses genes including two *LBD* genes. Moreover, we showed that IAA15 interacted with ARF7 and ARF19 and bound to the promoters of *LBD16* and *LBD29*.

## Materials and Methods

### Plant Materials and Growth Conditions

The *Arabidopsis thaliana* lines used in this study were in the Columbia background. For surface sterilization, seeds were soaked for 1 min in 70% EtOH, followed by 10 min in 1/10-diluted commercial bleach (0.4% NaOCl) and three washes with sterile distilled water. Surface-sterilized seeds were sown on agar plates containing half-strength Murashige-Skoog (MS) salts and vitamins (Murashige and Skoog, 1962), 2.0% sucrose, and 0.8% agar. The plates were incubated for 3 d at 4°C in the dark, and then at 22°C in a growth chamber under a 16 h light/8 h dark photoperiod with a light intensity of ~120 μmol m^-2^ s^-1^. Ten- to twelve-day-old seedlings were transferred to soil and grown under the same conditions. To explore the plant response to exogenous auxin, IAA15^P75S^ OX plants were vertically germinated and grown on MS medium containing 0, 10, 25, or 50 nM NAA with or without 50 μM DEX and photographed at 14 d after germination. For stress treatments, IAA15^P75S^ OX plants were vertically germinated and grown on MS medium containing 50 μM DEX plus NaCl (50 or 100 mM), mannitol (200 or 300 mM), or ABA (0.1 or 0.2 μM). The response to stress was estimated by measuring primary root length at 14 d after germination.

### Plasmid Construction, Site-Directed Mutagenesis, and Plant Transformation

To generate the *DEX_pro_*::*3XFLAG*-*IAA15^WT^* and *DEX_pro_*::*3XFLAG*-*IAA15^P75S^* constructs, the coding sequence of *IAA15* was amplified by PCR from a cDNA library prepared from Arabidopsis seedlings using gene-specific primers (**Supplementary Table 1**) and cloned into the T-blunt vector (Solgent, Korea). The amino acid substitution (Pro-75 to Ser-75) in full-length IAA15 was produced with the primers listed in **Supplementary Table 1** using a QuikChange Site-directed Mutagenesis kit (Stratagene, USA). *IAA15^WT^* and *IAA15 ^P75S^* were subcloned into the *Bam*HI and *Spe*I sites of the pBlueScript II KS (+) vector containing *3XFLAG*. *3XFLAG*-*IAA15^WT^* and *3XFLAG*-*IAA15^P75S^* were cloned into the pTA7002 binary vectors under the control of DEX-inducible promoter. For *IAA15_pro_*::*GUS*, a 2.4 kb genomic region upstream of the ATG start codon was cloned into the *Hin*dIII and *Sal*I sites of pBlueScript II KS (+) containing *GUS*. *IAA15_pro_*::*GUS* was cloned into the *Hin*dIII and *Sac*I sites of the pPZP211 binary vector. All constructs were confirmed by sequencing and transformed into Arabidopsis (Col-0) using *Agrobacterium* strain GV3101 (pMP90) by the floral dip method (Clough and Bent, 1998). Transformants were selected on MS medium containing the proper antibiotics. T_3_ homozygous progeny of transgenic plants expressing high levels of *IAA15* were used for all experiments.

### Extraction and Immunoblot Analysis of Arabidopsis Proteins

To extract total plant proteins, tissues were ground in liquid nitrogen and extracted in protein extraction buffer (50 mM HEPES, pH 7.5, 5 mM EDTA, 5 mM EGTA, 1 mM Na_3_VO_4_, 25 mM NaF, 50 mM-glycerophosphate, 2 mM DTT, 2 mM PMSF, 5% glycerol, 1% Triton X-100, and protease inhibitor). After two rounds of centrifugation at 12,000 × g for 10 min, the supernatants were transferred to clean tubes and stored at -80°C until use. Protein concentrations were determined using a Bio-Rad Protein Assay kit (Bio-Rad) with BSA as a standard. For immunoblot analysis, 50 µg total protein samples were separated by 10% SDS-PAGE and transferred to PVDF membranes. Proteins were detected using mouse anti-Flag (1:5,000; Sigma, USA) as a primary antibody and horseradish peroxidase-conjugated anti-mouse as a secondary antibody (1:5,000) and visualized using an ECL kit (Bio-Rad, USA).

### Histochemical GUS Assays

To investigate the expression pattern of *IAA15*, GUS activity in *IAA15_pro_*::*GUS* plants was detected histochemically as described by Jefferson et al. (1987) with slight modifications. Briefly, the tissue was incubated in 2 mM 5-bromo-4-chloro-3-indolyl- β-D-glucuronic acid in 50 mM phosphate buffer, pH 7.0, containing 0.5 mM K_3_Fe(CN)_6_ and 0.5 mM K_4_Fe(CN)_6_ for 6 h at 37°C. The tissue was rinsed with 50 mM phosphate buffer, fixed, and cleared overnight in ethanol (100%): acetic acid (9:1, v/v) at room temperature. The sample was photographed under a Nikon SMZ1000 stereoscopic microscope equipped with an OLYMPUS C-5050 ZOOM digital camera.

### *In Vivo* Turnover and Ubiquitination Assays

For the *in vivo* turnover assay of IAA15 proteins, 10-day-old seedlings overexpressing IAA15^WT^ or IAA15^P75S^ were pretreated with 50 μM DEX and 10 μM MG132 for 24 h, and then treated with 10 μM NAA in the presence of 1 mM cycloheximide (CHX) for 4 h. 50 µg total protein samples were separated by SDS-PAGE, and immunoblot analysis was performed using mouse anti-Flag (Sigma, USA). For the ubiquitination assay, 10-day-old DEX-treated IAA15^WT^ OX and IAA15^P75S^ OX seedlings were pretreated with 10 μM MG132 for 2 h, and then treated with or without 10 μM NAA for 24 h. Total plant protein (1 mg) was immunoprecipitated using anti-Flag antibody coupled to agarose beads (Sigma, St. Louis, MO, USA) for 4 h at 4°C. The beads were recovered by centrifugation, washed five times with extraction buffer, and eluted with 30 μL SDS-PAGE sample buffer. The eluted samples were separated by SDS-PAGE, and immunoblot analysis was performed using a monoclonal anti-ubiquitin antibody (Santa Cruz Biotechnology, Santa Cruz, CA, USA) as described (Miura et al., 2011).

### Quantitative RT-PCR

Total RNA was extracted from the samples using an RNA purification kit (Macherey-Nagel, Germany), and 5 μg total RNA was reverse transcribed in a 100 μl reaction volume using SuperScript II RNase-Reverse Transcriptase (Invitrogen, USA). Quantitative RT-PCR was performed in a 10 μl reaction volume containing 1 μl RT products, 10 pmol of gene-specific primers, and 5 μl SsoFast EvaGreen Supermix (Bio-Rad, USA) using the CFX384 Real-Time System (Bio-Rad, USA). The reaction conditions included an initial 5 min pre-incubation at 94°C, 45 cycles of 94°C for 30 s, 55°C for 30 s, and 72°C for 40 s, followed by melting curve analysis *via* 90 cycles at 55°C, increasing by 0.5°C/cycle, and a final cooling step for 10 min at 72°C. The primers used for PCR are shown in **Supplementary Table 2**.

### Yeast Two-Hybrid Analysis

The full-length open reading frames of *IAA15* and *ARF7*/*ARF29* were amplified using gene-specific primers (**Supplementary Table 1**) and cloned into pGAD424 (AD) and pAS2-1 (BD), respectively. The AD and BD plasmids were co-transformed into yeast strain pJ69-4A for interaction analysis, and colonies were selected after 2–3 days of growth at 30°C on SD-LEU/TRP medium. The transformants were tested for positive bait-prey interactions by monitoring the activation of HIS3 reporter genes, as described in the Clontech yeast protocol handbook.

### Chromatin Immunoprecipitation (ChIP) Assay

Two-week-old IAA15^P75S^ OX seedlings were treated with or without 50 μM DEX for 24 h, and the samples were collected. ChIP was carried out as described (Bastow et al., 2004; Tian et al., 2005) using mouse polyclonal anti-Flag antibody (1:3,000; Sigma, USA). PCR amplification was performed quantitatively using the CFX384 Real-Time System (Bio-Rad, USA). The immunoprecipitation was replicated three times, and each sample was quantified at least in triplicate. The ChIP primers are listed in **Supplementary Table 2**.

## Results

### Transgenic Plants Expressing an IAA15 Gain-of-Function Mutant Show Defects in Lateral Root Development

**Figure 1:**
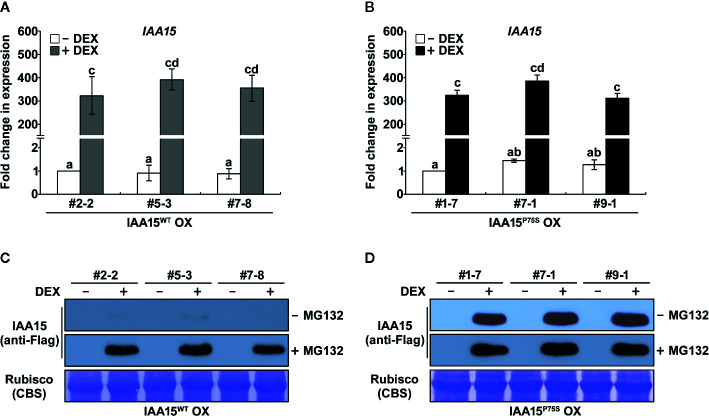
IAA15^P75S^ protein is stable. **(A, B)** Analysis of *IAA15* transcript levels *via* qRT-PCR using total RNA isolated from 2-week-old seedlings of three independent IAA15^WT^ OX **(A)** and IAA15^P75S^ OX **(B)** lines with or without 50 μM DEX induction of IAA15^WT^ and IAA15^P75S^ expression. The bars indicate the mean ± S.D. (*n* = 3). Different letters represent significant differences. **(C, D)** Immunoblot and Coomassie blue staining (CBS) of a polyacrylamide gel containing total proteins isolated from the IAA15^WT^ OX **(C)** and IAA15^P75S^ OX **(D)** seedlings with or without 50 μM DEX in the absence and presence of MG132. The gel blot was probed with anti-Flag antibody (anti-Flag). The Rubisco band detected by CBS shows the amount of protein loaded in each well.

Almost all Aux/IAA gain-of-function mutants having point mutation at the degron motif (VGWPP) of these proteins showed auxin deficient phenotypes (Reed, 2001). However, to date, a gain-of-function mutant of IAA15 has not been isolated. To identify the biological function of IAA15, in this study, we constructed transgenic plants overexpressing wild-type IAA15 (IAA15^WT^) and a gain-of-function mutant of IAA15 (IAA15^P75S^) under the control of the *35S* promoter. IAA15^P75S^ contains a Pro-75 to Ser-75 substitution in domain II of IAA15. Three independent lines of homozygous transgenic plants overexpressing IAA15^WT^ (IAA15^WT^ OX) were selected (**Supplementary Figure 1**). However, we failed to generate homozygous transgenic plants overexpressing IAA15^P75S^ (IAA15^P75^ OX), perhaps because they were all embryonically lethal. Therefore, we generated transgenic plants expressing IAA15^WT^ and IAA15^P75S^ under the control of a DEX-inducible promoter. Three independent lines of homozygous transgenic plants with high levels of IAA15 transcripts were selected after DEX treatment (**Figure 1**).

**Figure 2:**
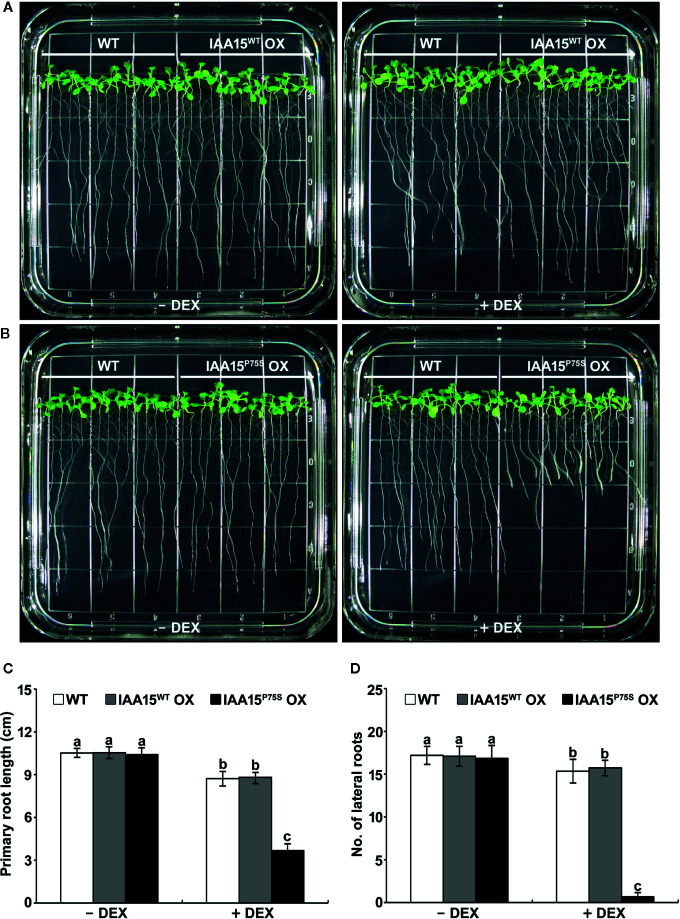
Primary root growth and lateral root formation are inhibited in IAA15^P75S^ OX plants. **(A, B)** Root growth phenotypes of wild-type (WT), IAA15^WT^ OX **(A),** and IAA15^P75S^ OX **(B)** with and without DEX. Shown are two-week-old seedlings grown on MS medium with or without 50 μM DEX. **(C, D)** Primary root length **(C)** and lateral root number **(D)** of 2-week-old seedlings of IAA15^WT^ OX and IAA15^P75S^ OX grown on MS medium with or without 50 μM DEX. The bars indicate the mean ± S.D. (*n* = 20 to 25). Different letters indicate significant differences (P < 0.05) among lines that were explored through Tukey’s multiple comparisons tests.

We investigated the auxin-related phenotypes of these transgenic plants. The treatment of IAA15^WT^ OX plants with or without DEX showed similar phenotypes to WT in terms of plant growth and development (**Figures 2A, C, D** and **Supplementary Figure 2**). DEX-untreated IAA15^P75S^ OX plants also showed no different phenotypes compared to WT, while DEX-treated IAA15^P75S^ OX plants showed defects in root development (**Figures 2B–D** and **Supplementary Figure 3**). In particular, the primary root length and lateral root number in DEX-treated IAA15^P75S^ OX plants were reduced by approximately 40% and 90%, respectively, compared to the WT (**Figures 2C, D**), suggesting that the IAA15^P75S^ protein is negatively involved in primary and lateral root development. Because each of three independent lines of IAA15^WT^ OX and IAA15^P75S^ OX showed similar expression patterns and root growth phenotype, we mixed seeds from these lines together and used these for further studies, and hereafter refer as IAA15^WT^ OX and IAA15^P75S^ OX, respectively.

### *IAA15* Is Expressed During Lateral Root Initiation and Development

**Figure 3:**
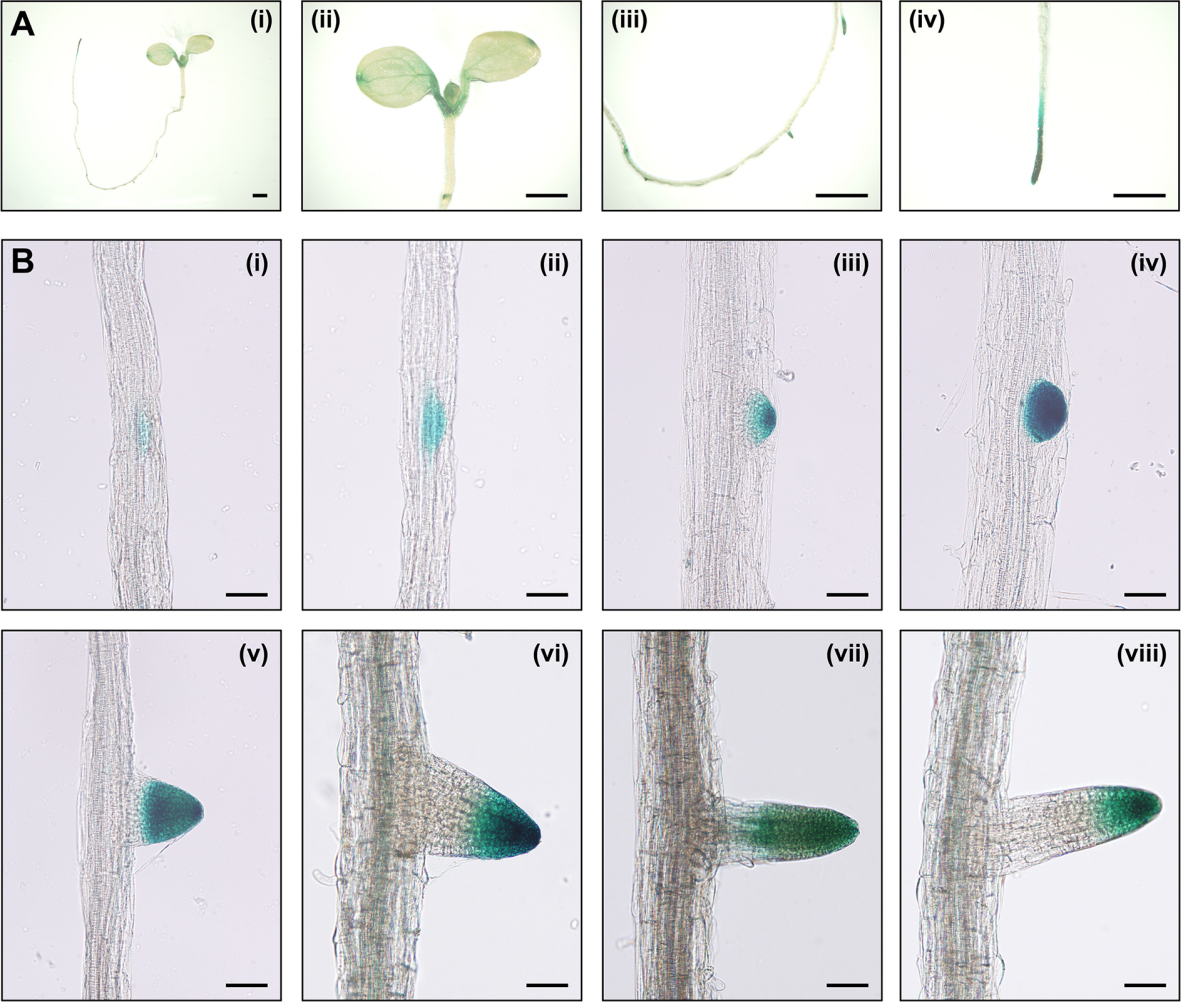
The expression patterns of *IAA15* in Arabidopsis tissues. **(A)** Tissue-specific expression of *IAA15* in *IAA15_pro_*::*GUS* transgenic plants, including a 7-day-old seedling (i), cotyledons, and shoot apical region (ii), lateral root and primordia in the root elongation zone (iii), and primary root tip (iv). Bars = 1 mm. **(B)** Expression pattern of *IAA15* during different stages of lateral root formation. Seven-day-old seedlings were incubated for 24 h in 5-bromo-4-chloro-3-indolyl glucuronide (X-gluc) for GUS staining. Bars = 50 μm.

The investigation of the expression patterns is helpful in estimating the unknown physiological function of a specific gene. To explore the expression pattern of *IAA15*, we generated transgenic plants expressing the *β*-*glucuronidase* (*GUS*) reporter gene under the control of the *IAA15* promoter. GUS activities were detected in the shoot apical meristem, petioles, veins, tips of cotyledons, primary root tips, lateral root primordia, and lateral root tips (**Figure 3A**). GUS activities were also highly detected at all stages in morphologically recognizable lateral root primordia and highly in primordium tips after maturation (**Figure 3B**). To examine the presence of specific *cis*-regulatory elements in the promoter, we performed in silico analysis on the *IAA15* promoter. We found that *IAA15* promoter includes many root-specific *cis*- regulatory elements, such as Sorlip1, AS1-Box, and FaRB7_root-specific (**Supplementary Figure 4**) (Puzio et al., 2000; Jiao et al., 2005; Vaughan et al., 2006; Kakrana et al., 2017). These *cis*-elements were also found on the *IAA14* gene promoter, which plays role in lateral root development. Taken together, these results suggest that IAA15 plays a functional role in lateral root development.

### Mutation of the Degron Motif of IAA15 Increases Protein Stability by Inhibiting Poly-Ubiquitination

Aux/IAAs are short-lived proteins that are rapidly degraded by the ubiquitin-proteasome mediated pathway (Santner et al., 2009). Gain-of-function mutations of Aux/IAA genes confer auxin-related phenotypes by stabilizing Aux/IAA proteins (Reed, 2001). As expected, IAA15 proteins were not detected in IAA15^WT^ OX plants because this protein is unstable in normal condition (**Figure 1C** and **Supplementary Figure S1**). By contrast, IAA15 highly accumulated in response to treatment with the proteasome inhibitor MG132. Interestingly, high levels of IAA15 were also detected in IAA15^P75S^ OX plants in response to DEX treatment, even in the absence of MG132 (**Figure 1D**). This result indicates that IAA15^P75S^ become stable in normal growth condition.

**Figure 4:**
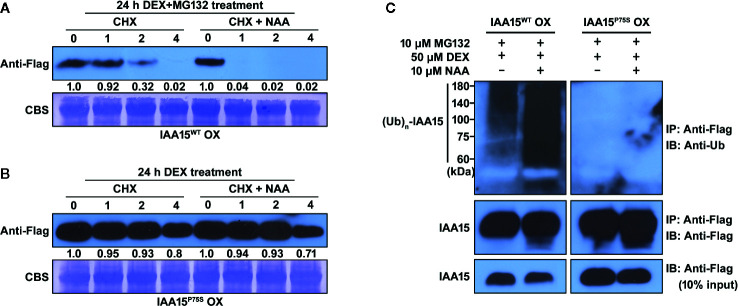
IAA15^P75S^ protein has greater stability than IAA15^WT^. **(A, B)** Stability of IAA15^WT^ and IAA15^P75S^ *in vivo*. To induce accumulation of IAA15^WT^ and IAA15^P75S^, 14-day-old seedlings of IAA15^WT^ OX **(A)** and IAA15^P75S^ OX **(B)** were treated with 50 μM DEX and 10 μM MG132 for 24 h, followed by 1 mM cycloheximide (CHX) with or without 10 μM NAA. Images show polyacrylamide gels of total proteins isolated from the seedlings after 0, 1, 2, and 4 h of CHX or CHX + NAA treatment. IAA15^WT^ and IAA15^P75S^ were detected with anti-Flag antibody. Rubisco band detected by CBS shows the amount of protein loaded in each well. **(C)** Auxin-dependent poly-ubiquitination of IAA15^WT^ and IAA15^P75S^. IAA15 poly-ubiquitination was detected by immunoblotting (IB) with anti-ubiquitin antibody after immunoprecipitation (IP) with anti-Flag antibody from DEX-treated IAA15^WT^ OX and IAA15^P75S^ OX plants treated with MG132 in the absence or presence of NAA (upper panel). IAA15 proteins were detected by immunoblotting with anti-Flag antibody (lower panel, 10% input).

To confirm whether IAA15^P75S^ is indeed more stable than IAA15^WT^, we measured the degradation rate of these proteins in IAA15^WT^ OX and IAA15^P75S^ OX plants. We accumulated IAA15^WT^ and IAA15^P75S^ proteins in these transgenic plants by treatment with MG132 and DEX for 24 h, and then measured the amount of proteins by Western blots in the presence or absence of NAA plus CHX for 4 h. CHX was used to prevent *de novo* synthesis of proteins in plants. As a result, IAA15^WT^ was rapidly degraded within 2 h by the treatment with or without NAA and almost undetectable after 4 h (**Figure 4A**). In contrast, IAA15^P75S^ was stable even in the presence of NAA (**Figure 4B**), indicating that IAA15^P75S^ is more stable than IAA15^WT^.

Next, to examine whether the Pro-75 to Ser point mutation of the degron domain would affect the poly-ubiquitination of IAA15 by the SCF^TIR1/AFBs^ complex, we measured the ubiquitination efficiencies of IAA15^WT^ and IAA15^P75S^ proteins. To block the auxin-induced degradation of ubiquitinated IAA15, we treated DEX-pretreated IAA15^WT^ OX and IAA15^P75S^ OX plants with MG132 in the absence or presence of NAA. The accumulated IAA15 proteins were immunoprecipitated from total extracts of IAA15^WT^ OX and IAA15^P75S^ OX plants with anti-Flag antibody and immunoblotted with anti-ubiquitin antibody to measure the levels of ubiquitinated IAA15 protein, as described (Miura et al., 2011). Inputs of IAA15 proteins in IAA15^WT^ OX and IAA15^P75S^ OX plants were similarly detected by treatments with DEX and MG132 (**Figure 4C**, lower panel). As expected, poly-ubiquitinated IAA15^WT^ was highly detected in IAA15^WT^ OX plant by treatment with NAA, while those of IAA15^P75S^ were less detected in IAA15^P75S^ OX plant in both the presence and the absence of NAA (**Figure 4C**, upper panel). Taken together, these results suggest that the point mutation in the degron motif of IAA15 increases its protein stability by the inhibition of poly-ubiquitination and the subsequent proteasome-mediated degradation.

### IAA15 Negatively Regulates Auxin-Responsive Genes Including *LBD16* and *LBD29*

To examine the effect of stabilization of IAA15 protein on auxin response, we analyzed root growth in IAA15^P75S^ OX plants in response to exogenous auxin (**Supplementary Figure 4**). As expected, DEX-untreated IAA15^P75S^ OX seedlings showed inhibited primary root growth and increased lateral root number in response to 10 and 25 nM NAA. In DEX-treated IAA15^P75S^ OX seedlings, however, the primary root growth was only slightly reduced, and there was no significant difference in lateral root number in the presence of 10 and 25 nM NAA. This insensitivity of IAA15^P75S^ OX seedlings to auxin was no longer detected in a high auxin level (over 50 nM) (**Supplementary Figure 4**). These results indicate that the stabilized IAA15 protein represses auxin response.

**Figure 5:**
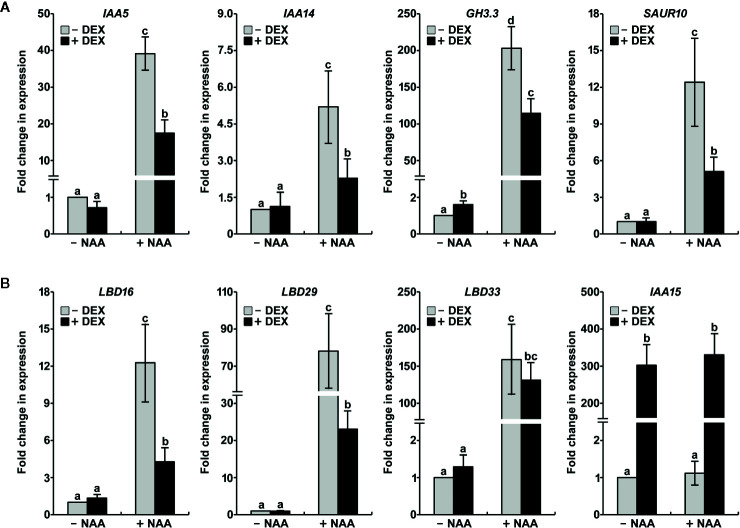
IAA15^P75S^ inhibits auxin-responsive gene expression. Total RNA was extracted from IAA15^P75S^ OX plants treated with or without 10 μM NAA and/or 50 μM DEX. **(A)** Transcript levels of auxin-responsive genes are reduced in IAA15^P75S^ OX plants. *IAA5*, *IAA14*, *GH3.3*, and *SAUR10* transcript levels were measured by qRT-PCR using specific primers. The bars indicate the mean ± S.D. (*n* = 3). Different letters indicate significant differences (P < 0.05) among lines that were explored through Tukey’s multiple comparisons tests. **(B)** The mutation of IAA15 inhibits the expression of lateral root-related *LBDs*. *IAA15*, *LBD16*, *LBD29*, and *LBD33* transcript levels were measured by qRT-PCR using specific primers. The bars indicate the mean ± S.D. (*n* = 3). Different letters indicate significant differences (P < 0.05) among lines that were explored through Tukey’s multiple comparisons tests.

Because the stabilization of IAA15 resulted in impairment of auxin-related phenotypes, we hypothesized that the accumulation of IAA15 inhibits the expression of auxin-responsive genes. To test this hypothesis, DEX-untreated and -treated IAA15^P75S^ OX plants have analyzed the expression of auxin-responsive genes such as *IAA5*, *IAA14*, *GH3.3*, and *SAUR10* in the absence and presence of NAA. The expression of these genes was enhanced by treatment with NAA alone, while the induction of these genes by NAA was significantly suppressed by the accumulation of IAA15^P75S^ (**Figure 5A**). In addition, we also analyzed the expression of *LBD* genes, which are critical regulators of lateral root formation. As expected, *LBD16*, *LBD29*, and *LBD33* were highly induced by NAA treatment (**Figure 5B**). By the accumulation of IAA15^P75S^, however, the inductions of *LBD16* and *LBD29* genes were significantly reduced, and that of *LBD33* was slightly reduced (**Figure 5B**). These results indicate that the gain-of-function mutation of IAA15 suppresses the transcription of auxin-responsive genes including *LBD16* and *LBD29*.

### IAA15 Binds to the Promoters of *LBD16* and *LBD29*

**Figure 6:**
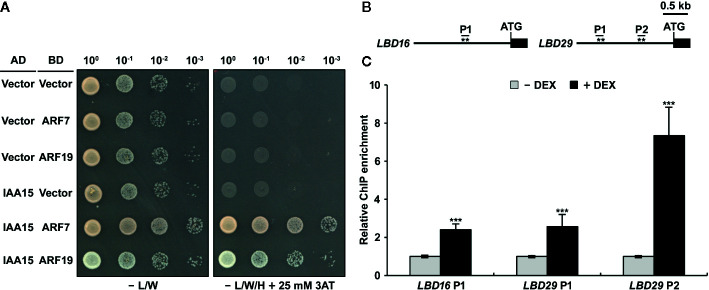
IAA15 interacts with ARF7 and ARF19 and binds to the promoters of *LBD16* and *LBD29*. **(A)** Yeast two-hybrid analysis showing the interaction of IAA15 with ARF7 and ARF19. Various combinations of plasmids were co-transformed into yeast strain pJ69-4A. Shown are growth tests of the transformants on selective SD medium lacking Leu and Trp (-L/W) (control) and on SD medium lacking Leu, Trp, and His (-L/W/H) supplemented with 25 mM 3-amino-1,2,4-triazole (3-AT) to demonstrate activation of the HIS3 reporter gene. **(B)** Diagram of the *LBD16* and *LBD29* promoters, which contain target sequences of ARF7 and ARF19. The asterisks (**) show the positions of primers used for ChIP-qPCR. **(C)** ChIP assay of the binding of IAA15 to the ARF7- and ARF19-response elements in the *LDB16* and *LBD29* promoters *in vivo*. ChIP assays were performed with chromatin prepared from IAA15^P75S^ OX treated with or without 50 μM DEX by using anti-Flag antibody. ChIP-DNA was applied to RT-qPCR using primers specifically targeting to promoter regions of *LBD16* and *LBD29*. The bars indicate the mean ± S.D. (*n* = 3). Significant differences were analyzed by Student’s *t*-test (***P < 0.01).

Although IAA15 negatively regulates the expression of *LBD16* and *LBD29*, it is likely to repress auxin-responsive transcription by interacting with the partner ARF proteins because Aux/IAA repressors do not have a DNA-binding domain (Guilfoyle, 2015; Powers and Strader, 2020). To know the molecular mechanism to explain how IAA15 inhibits the expression of *LBD16* and *LBD29* genes, we first investigated whether IAA15 interacts with ARF7 or ARF19, which are direct and positive regulating transcription factors in the expression of *LBD16* and *LBD29* genes (Okushima et al., 2007). IAA15 was previously shown to interact with the C-terminal fragments (Domain III and IV) of ARF7 and ARF19 in a yeast two-hybrid assay (Vernoux et al., 2011). Therefore, we examined the interactions of IAA15 with the full-length ARF7 and ARF19 in yeast cells. As expected, IAA15 physically interacts with full-length ARF7 and ARF19 (**Figure 6A**).

The previous investigation reported that ARF7 and ARF19 directly bind to auxin-responsive elements (AuxREs) of *LBD16* and *LBD29* promoters. (**Figure 6B**) (Okushima et al., 2007). To explore whether IAA15 binds to these AuxREs of *LBD* promoters, therefore, we performed a chromatin immunoprecipitation (ChIP) assay in DEX-untreated and -treated IAA15^P75S^ OX plants using anti-Flag antibody. The enrichment of these AuxREs was highly increased by the induction of IAA15^P75S^ (**Figure 6C**). These results strongly suggest that IAA15 binds to the promoters of *LBD16* and *LBD29*, presumably indirectly through interactions with an ARF7 and ARF19.

## Discussion

### The Stabilized IAA15 Inhibits the Lateral Root Growth

Almost all gain-of-function mutations of *Aux*/*IAA* occurring within conserved domain II result in defective plant growth due to the stabilization of Aux/IAA. In the current study, we found that IAA15^P75S^ OX plants exhibited auxin-related abnormal developmental phenotypes such as short primary roots and reduced lateral root formation (**Figure 2** and **Supplementary Figures 2** and **3**). These developmental phenotypes of IAA15^P75S^ OX plants were caused by the inhibition of auxin signaling through an accumulation of IAA15^P75S^ (**Figures 1** and **5** and **Supplementary Figure 4**). The accumulation of IAA15^P75S^ was due to the inhibited poly-ubiquitination by the ubiquitin E3 ligase complex (**Figure 4**). Interestingly, these auxin-related root defective phenotypes of IAA15^P75S^ OX plants are also found in gain-of-function mutants of other *Aux*/*IAAs* such as *iaa3*/*shy2*, *iaa14*/*slr1-1*, *iaa19/msg2*, and *iaa28* (Reed, 2001; Liscum and Reed, 2002; Mockaitis and Estelle, 2008). The similar root phenotypes of those mutants suggest that several Aux/IAA proteins may be redundantly involved in plant root development. The next possible speculation is that similar root defective phenotypes by several gain-of-function mutants can be caused by over-accumulation of corresponding Aux/IAA proteins. Over-accumulated Aux/IAA proteins in those mutants may cause non-specific interactions with ARF partner proteins, which result in unusual auxin-related root development phenotypes. To determine which *Aux*/*IAA* genes are *bona* *fide* involved in root development, the more detailed experimental investigations by using new transgenic plants expressing AUX/IAAs mutant under the control of their promoters and loss-of-function mutant of Aux/IAAs should be performed.

### IAA15 Is a Component of Aux/IAA-ARF Modules Inhibiting Lateral Root Formation

The molecular model explaining the mechanism of lateral root formation is well established based on many studies (Tian and Reed, 1999; Rogg et al., 2001; Tatematsu et al., 2004; Okushima et al., 2005; Weijers et al., 2005; Wilmoth et al., 2005; Fukaki et al., 2006; Li et al., 2006). According to this model, auxin de-represses the activity of the transcriptional activators ARF7 and ARF19 through the degradation of Aux/IAAs such as IAA3, IAA14, IAA19, and IAA28, which directly activates the transcription of downstream genes including *LBD16* and *LBD29* to induce lateral root formation (Okushima et al., 2007). The lateral root formation is redundantly regulated by multiple Aux/IAA-ARF modules such as IAA3/SHY2-ARFs, IAA14/SLR-ARF7 and -ARF19, IAA19/MSG2-ARF7, and IAA28-ARFs. In the current study, we found that in addition to other IAAs, IAA15 also negatively regulates lateral root formation through the transcriptional repression of *LBD16* and *LBD29* (**Figures 1** and **4**). In addition, we showed that IAA15 directly interacts with ARF7 and ARF19 and binds to the promoters of *LBD16* and *LBD29*. These findings suggest that the roles of the IAA15-ARF7 and -ARF19 modules in lateral root development are similar to those of other known IAA-ARF modules.

The identification of several IAA-ARF modules with redundant roles in lateral root formation raises the question of why multiple Aux/IAAs redundantly regulate this process. We propose two possibilities. First, several Aux/IAA-ARF modules could be specifically involved in various stages of lateral root formation. Lateral root formation is a complex process involving three major steps: lateral root initiation, lateral root primordia development, and lateral root emergence (Malamy and Benfey, 1997; Jung and McCouch, 2013). For example, IAA28-ARF modules are involved in the specification of lateral root founder cells in the basal root meristem during the early stage of lateral root formation (De Rybel et al., 2010). IAA14/SLR-ARF7 or -ARF19 modules are involved in the asymmetric division of lateral root founder cells for lateral root initiation (Fukaki et al., 2002; Okushima et al., 2005; Okushima et al., 2007). The IAA12/BDL-ARF5 module also participates in lateral root initiation, and IAA3/SHY2-ARF modules regulate lateral root primordia development and emergence after lateral root initiation (De Smet et al., 2010). IAA15-ARF7 or -ARF19 modules may be involved in specific stages of lateral root formation. However, IAA15-ARF modules may be involved in all stages of this process because *IAA15* is expressed in all stages in lateral root initiation and development. Second, specific Aux/IAA-ARF modules could be responsive to particular environmental stresses. Abiotic stresses such as salt stress, drought stress, and nutrient depletion affect root development, including lateral root formation (Xiong et al., 2006; Zolla et al., 2010; Sun et al., 2017; Huang et al., 2018). In response to specific environmental stresses, specific Aux/IAA may be stabilized to inhibit lateral root formation, which contributes plants to survive. This hypothesis may explain how Aux/IAAs regulate growth and defense trade-off in plants. In the future, detailed studies elucidating the biological functions of IAAs under stress conditions should be performed.

## Data Availability Statement

The original contributions presented in the study are included in the article/**Supplementary Material**; further inquiries can be directed to the corresponding author.

## Author Contributions

SK and WC designed, planned, and organized the experiments. SK, SB, JA, SH, NN, and HD generated all Arabidopsis transgenic lines and all plant materials used in this study. SK, SB, JA, and JH performed the research. SK, J-YK, JH, and WC wrote the article.

## Funding

This work was supported by a grant from the Next-Generation BioGreen 21 Program (No. PJ01325401) funded by the Rural Development Administration, by the Basic Science Research Program through the National Research Foundation of Korea (NRF) funded by the Ministry of Education (No. 2018R1A6A3A11042628 and 2020R1A6A1A03044344), by a National Research Foundation of Korea (NRF) grant funded by the Korean government (MSIP) (No. 2019R1A2C1009932).

## Conflict of Interest

The authors declare that the research was conducted in the absence of any commercial or financial relationships that could be construed as a potential conflict of interest.
