# Supplementary material for: A Gain-of-Function Mutant of IAA15 Inhibits Lateral Root Development by Transcriptional Repression of LBD Genes in Arabidopsis
Source: Front Plant Sci. 2020 Aug 12;11:1239. doi: 10.3389/fpls.2020.01239 (PMC7434933; doi:10.3389/fpls.2020.01239)
Supplement: Supplementary file 1 [file DataSheet_1.docx]

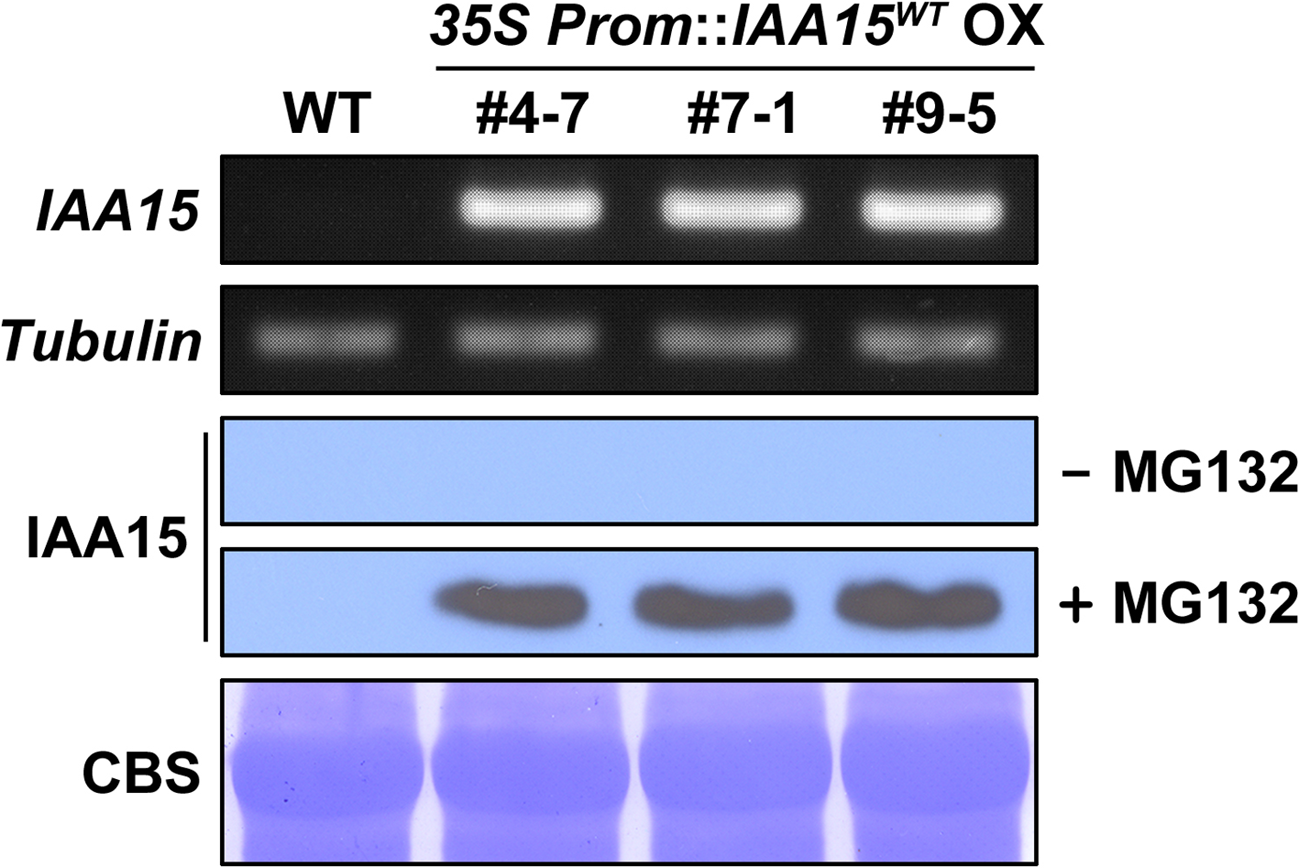


**Supplementary Figure 1** IAA15 protein degradation is dependent on the 26S proteasome pathway. *IAA15* and *Tubulin* (loading control) transcript levels were analyzed by RT-PCR using total RNA prepared from 3-week-old WT seedlings and seedlings from three independent IAA15^WT^ OX lines. Flag-tagged IAA15 protein levels were measured in transgenic plants in the absence and presence of MG132. IAA15 protein levels were detected by immunoblotting with anti-Flag antibody. The Rubisco band detected by CBS shows the amount of protein loaded in each well.


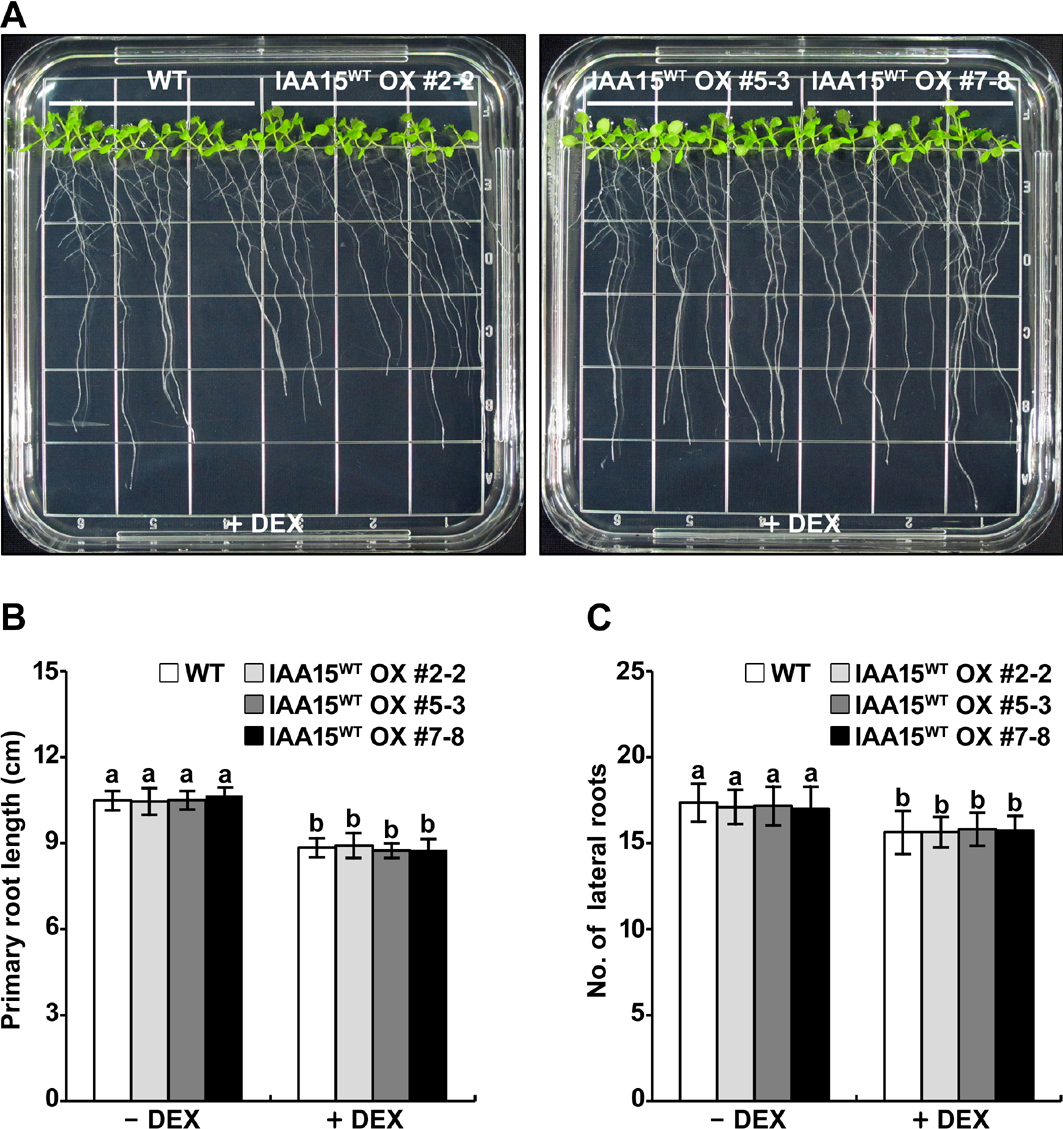


**Supplementary Figure 2** Three independent IAA15^WT^ OX lines show normal root growth in the presence of DEX. **(A)** Root growth in WT and three independent IAA15^WT^ OX lines. Two-week-old seedlings were grown vertically on MS medium with or without 50 μM DEX. **(B, C)** Primary root length and lateral root number were measured in 2-week-old seedlings grown on MS medium with or without 50 μM DEX. The bars indicate the mean ± S.D. (*n* = 20 to 25). Different letters indicate significant differences (*P* < 0.05) among lines that were explored through Tukey's multiple comparisons tests.


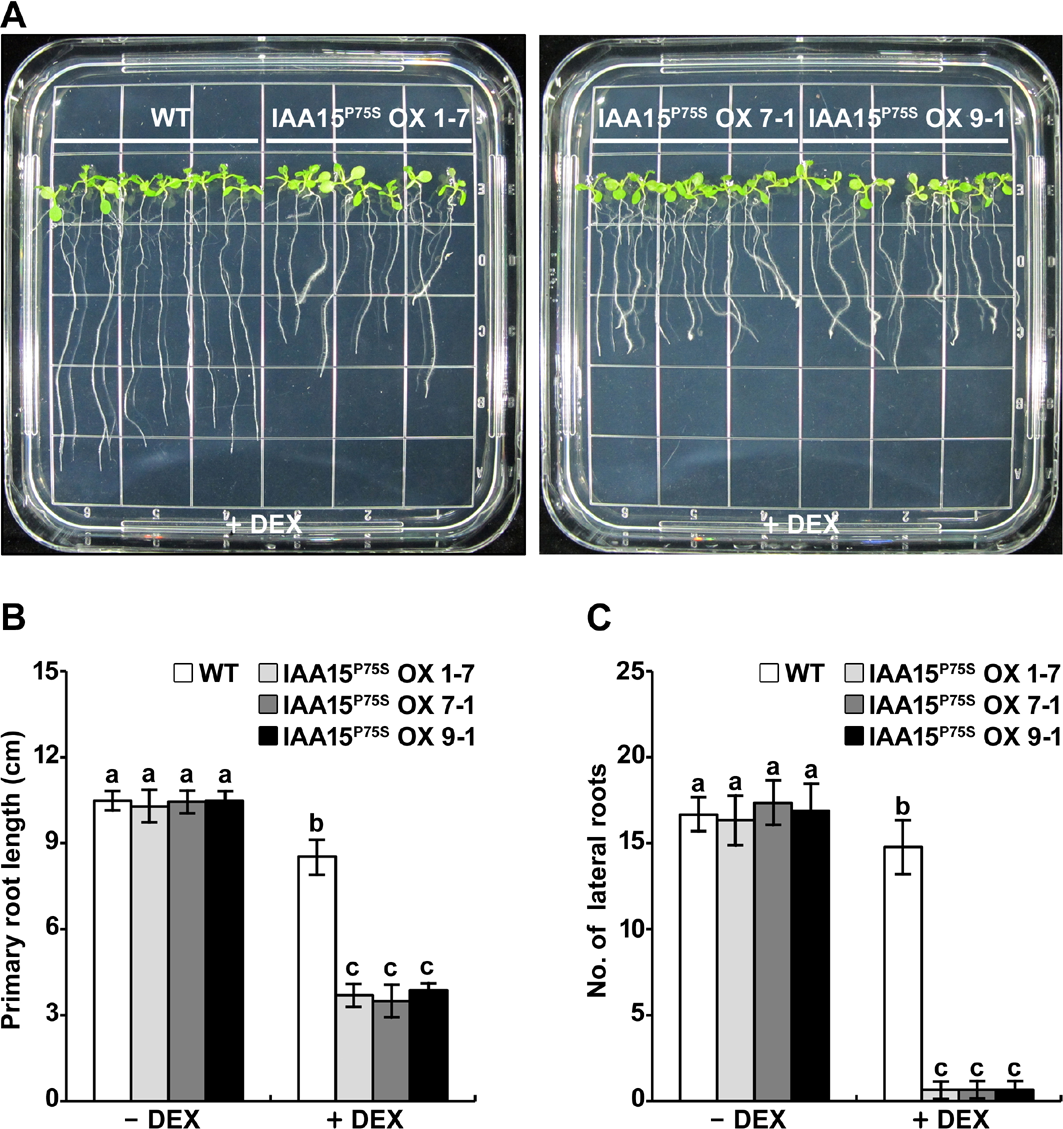


**Supplementary Figure 3** Three independent IAA15^P75S^ OX lines show the defective root growth in the presence of DEX. **(A)** Root growth in WT and three independent IAA15^P75S^ OX lines. Two-week-old seedlings were grown vertically on MS medium with or without 50 μM DEX. **(B, C)** Primary root length and lateral root number were measured in 2-week-old seedlings grown on MS medium with or without 50 μM DEX. The bars indicate the mean ± S.D. (*n* = 20 to 25). Different letters indicate significant differences (*P* < 0.05) among lines that were explored through Tukey's multiple comparisons tests.


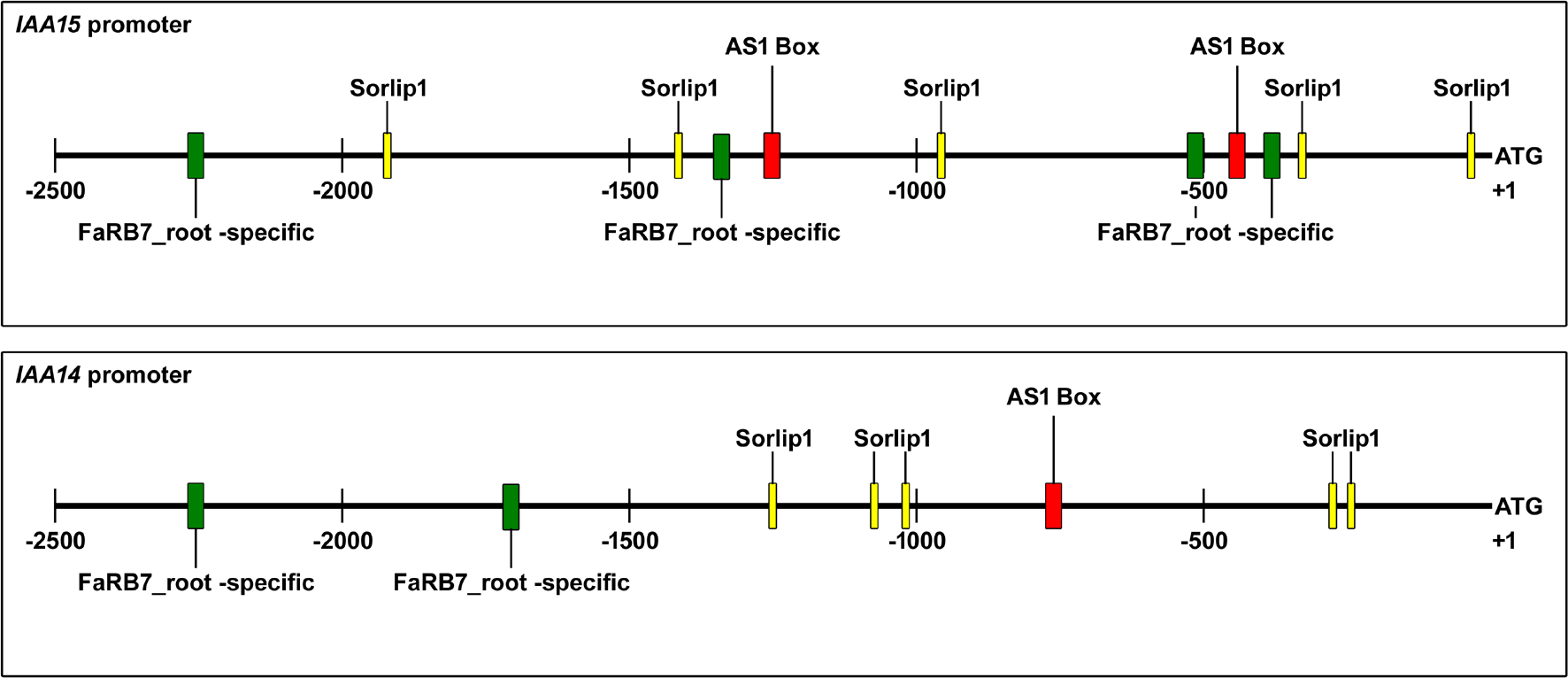


**Supplementary Figure 4** The promoters of *IAA15* and *IAA14* gene contain many root-specific *cis*-regulatory elements. The Sorlip 1 (AGCCAC), AS1 box (TGACGTCA), and FaRB7 (TTTCNTTTGG) are root-specific elements. The 2.5 kb region of each promoter was used for the profiling.


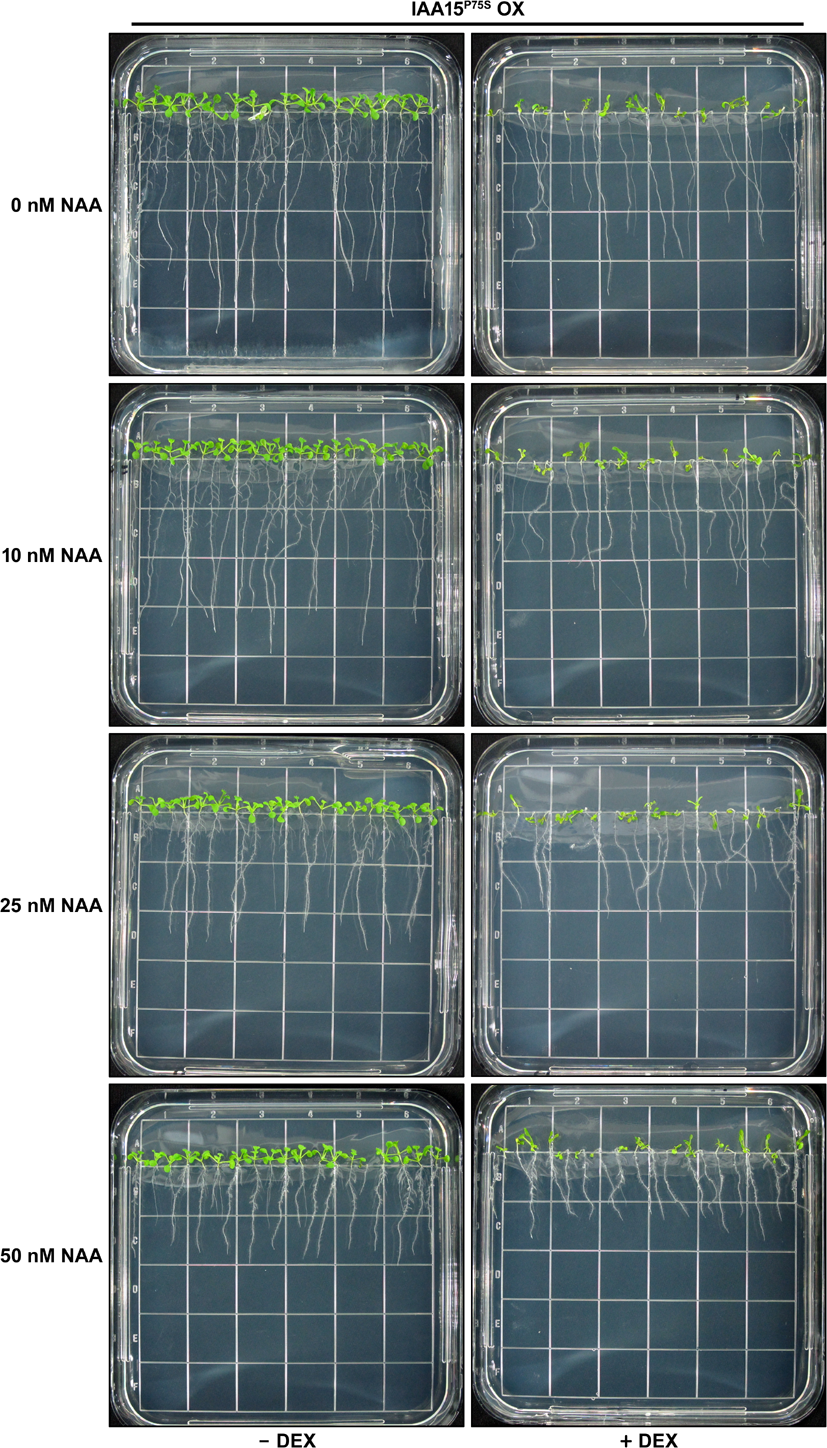


**Supplementary Figure 5** The sensitivity of plants to exogenous auxin is reduced by the accumulation of IAA15^P75S^. IAA15^P75S^ OX seedlings were grown vertically on MS medium with or without 50 μM DEX plus 0, 10, 25, or 50 nM NAA. The roots were photographed at 14 days after germination.

**Supplementary Table 1** Primers used for cloning and site-directed mutagenesis.

| **Construct** | **Position** | **Sequence** |
| --- | --- | --- |
| *IAA15* | F | 5'- ggatccATGTCACCGGAGGAATACGT -3' |
|  | R | 5'- actagtTCACTTACATATTGTTATTA -3' |
| *IAA15^P75S^*  (site-directed mutagenesis) | F | 5'- GTGGGCTGGTCGCCGGTAGCGACAGCGAGG -3' |
|  | R | 5'- CGCTACCGGCGACCAGCCCACCAACTGGTC -3' |
| *IAA15* promoter | F | 5'- aagcttCAGTAAATAGGGAGAAGACTG -3' |
|  | F | 5'- gtcgacGTTAGTTAACAACAAAAGTGC -3' |
| *IAA15*  (Gateway system for Y2H) | F | 5'- aaaaaagcaggcttaATGTCACCGGAGGAATACGT -3' |
|  | R | 5'- agaaagctgggtcTCACTTACATATTGTTATTA -3' |
| *ARF7*  (Gateway system for Y2H) | F | 5'- aaaaaagcaggcttaATGAAAGCTCCTTCATCA -3' |
|  | R | 5'- agaaagctgggtcTCACCGGTTAAACGAAGT -3' |
| *ARF19*  (Gateway system for Y2H) | F | 5'- aaaaaagcaggcttaATGAAAGCTCCATCAAAT -3' |
|  | R | 5'- agaaagctgggtcCTATCTGTTGAAAGAAGC -3' |

**Supplementary Table 2** Primers used for quantitative RT-PCR and ChIP-qPCR.

| **Construct** | **Position** | **Sequence** |
| --- | --- | --- |
| *IAA15*  (qRT-PCR) | F | 5'- AACGTAGATTCCTCGAGACC -3' |
|  | R | 5'- TCTAGAGCGGTGAAAAGCTG -3' |
| *IAA5*  (qRT-PCR) | F | 5'- AAGAGTCAAGTTGTGGGTTGGC -3' |
|  | R | 5'- AATGCAGCTCCATCTACACTCACT -3' |
| *IAA14*  (qRT-PCR) | F | 5'- GAATTCATGAACCTTAAGGAGACGGA -3' |
|  | F | 5'- CCCGGGTGATCTGTTCTTGAACTTCT -3' |
| *GH3.3*  (qRT-PCR) | F | 5'- ATGGAGGAGTCGTTGAACTCTGTG -3' |
|  | R | 5'- AAGCTCCATTATTGGCGTGAAACTC -3' |
| *SAUR10*  (qRT-PCR) | F | 5'- CGAAGTCGGTACATCGTTCCTATC -3' |
|  | R | 5'- CATGGAGATAAGAGACCTGAAGAAGA -3' |
| *LBD16*  (qRT-PCR) | F | 5'- GAGAGACTCATCATCAAACC -3' |
|  | R | 5'- CTAAGAGCCAAAGCCTGAAG -3' |
| *LBD29*  (qRT-PCR) | F | 5'- AAGTTCTGGGACGGTTCAAC -3' |
|  | R | 5'- GCTGATTGAAGCTCTTTGAG -3' |
| *LBD33*  (qRT-PCR) | F | 5'- ACCAATTTCGTCGACGAGAG -3' |
|  | R | 5'- TCCATGTTCTGGAGCCATTG -3' |
| *Tubulin*  (qRT-PCR) | F | 5'- CCAACAACGTGAAATCGACAG -3' |
|  | R | 5'- TCTTGGTATTGCTGGTACTCT -3' |
| *LBD16* promoter P1  (ChIP-qPCR) | F | 5'- AACCCAATAAATTAGAAGTC -3' |
|  | R | 5'- CAAAGGGTGTGAATGGTAGA -3' |
| *LBD29* promoter P1  (ChIP-qPCR) | F | 5'- CCTACGTACTGTTCAAATAT -3' |
|  | R | 5'- CTTTAACTTATATTTCTAAC -3' |
| *LBD29* promoter P2  (ChIP-qPCR) | F | 5'- TTCCACAACTGCTATGCGAT -3' |
|  | R | 5'- TGATATTTTCGTATCATTTA -3' |
